# Reply to Gijon-Nogueron, G. Comment on “Alfageme-Garcia et al. Respectful Children’s Shoes: A Systematic Review. *Children* 2024, *11*, 761”

**DOI:** 10.3390/children11101220

**Published:** 2024-10-08

**Authors:** Pilar Alfageme-García, Sonia Hidalgo-Ruiz, Sergio Rico-Martín, Julián Fernando Calderón-García, Víctor Manuel Jimenez-Cano, Juan Francisco Morán-Cortés, Belinda Basilio-Fernández

**Affiliations:** 1 Department of Nursing, University Centre of Plasencia, University of Extremadura, 10600 Plasencia, Spain; palfagemeg@unex.es (P.A.-G.) ; victormajc@unex.es (V.M.J.-C.); bbasfer@unex.es (B.B.-F.); 2 Department of Nursing, Nursing and Occupational Therapy College, University of Extremadura, 10003 Cáceres, Spain; sergiorico@unex.es (S.R.-M.); jfcalgar@unex.es (J.F.C.-G.)

After a hard effort to carry out this research, we are pleased to know that it causes great interest in society and in science. We are pleased to receive your feedback as it makes us better researchers. We will take it into account for the following investigations.

We would like to delve deeper into some of the points that have been raised:

First, this is a systematic review, which has taken us a long time to work on and we did not have much bibliography for it.

If the selected final projects follow a robust and rigorous methodology, their inclusion is justified. The validity of their results can be as high as that of the studies published in other academic journals. The final projects can address specific topics relevant to the systematic review that may not be widely covered in the published literature. This provides diversity and depth to the analysis, although we are aware of what you have said regarding the fact that they are not very scientific [1]. 

The final projects may contain original research and new data that are not available from other sources. Including these works in the systematic review provides a more complete and updated perspective, as well as expands the database and can help us avoid bias by considering a greater variety of sources. This contributes to a more comprehensive vision of the topic of study [1].

Secondly, the comment on the footwear article indicating that both respectful footwear and therapeutic footwear share the common objective of promoting the health and well-being of the feet. Both types of footwear are designed considering the biomechanics of the foot and seek to minimize podiatric problems. The scientific principles behind therapeutic footwear design, such as proper foot alignment, proper support, and pressure distribution, are relevant to respectful footwear. The findings and data from the therapeutic footwear studies provide valuable and applicable information [2].

Studies on therapeutic footwear often demonstrate specific foot health benefits, such as reducing pain, improving posture, and preventing injuries. These benefits are equally sought in the design of respectful footwear, which justifies the inclusion of these studies in the review. While respectful footwear focuses on the prevention of podiatric problems, therapeutic footwear focuses on the treatment. Integrating both perspectives can provide a more complete and holistic understanding of the impact of footwear on foot health [1].

Including articles on therapeutic footwear offers a more complete view of the needs and solutions in healthy footwear design. This allows the systematic review to address both the prevention and treatment of podiatric problems [1].

Findings from the studies on therapeutic footwear can be transferred and applied to the design and evaluation of respectful footwear [1]. For example, knowledge about how certain designs and materials affect foot health are directly relevant.

Thirdly, regarding the comment that explains how the Delphi method collects and synthesizes the opinions of experts in a specific field, say that including a Delphi study in a systematic review can provide deep and well-founded knowledge about the topic in question, based on experience and expert consensus [1]. Delphi studies are useful for identifying emerging trends, research priorities, and key areas of consensus in the literature. This can complement the empirical data collected in other types of studies, providing a more complete and holistic view. Delphi studies follow a structured and systematic process, including multiple rounds of questionnaires and controlled feedback. This methodological rigor ensures the validity and reliability of the results obtained. The Delphi method helps reduce individual and group biases using anonymous rounds and the aggregation of opinions. This can provide a balanced and objective assessment of the issues addressed [1].

The manuscripts used in this systematic review appear in verified databases, and you can review them in the following Table 1 databases:

The systematic review followed a clearly defined and structured methodology. This included the formulation of specific research questions, the definition of the inclusion and exclusion criteria, and the application of a pre-established protocol for the search and selection of studies [1].

The conclusions were derived from cohesive evidence, where multiple studies point in the same direction, reinforcing the validity of the findings.

With the limitations that we have found when working on this topic., it makes us aware of the importance of continuing research in this line.

## Figures and Tables

**Table 1:** Syntaxes of combined descriptors in the scientific database search [1].

Database.	Syntax Adopted
PubMed	‘Shoes AND Children AND Pediatrics’; ‘Shoes AND Children AND Children’s footwear’; ‘Shoes AND Children’s footwear AND Respectful children’s shoes’
Cochrane	‘Shoes AND Children AND Pediatrics’; ‘Shoes AND Children AND Children’s footwear’; ‘Shoes AND Children’s footwear AND Respectful children’s shoes’
Dialnet	‘Shoes AND Children AND Pediatrics’; ‘Shoes AND Children AND Children’s footwear’; ‘Shoes AND Children’s footwear AND Respectful children’s shoes’
Scopus	‘Shoes AND Children AND Pediatrics’; ‘Shoes AND Children AND Children’s footwear’; ‘Shoes AND Children’s footwear AND Respectful children’s shoes’
Web of Science	‘Shoes AND Children AND Pediatrics’; ‘Shoes AND Children AND Children’s footwear’; ‘Shoes AND Children’s footwear AND Respectful children’s shoes’
PsycINFO	‘Shoes AND Children AND Pediatrics’; ‘Shoes AND Children AND Children’s footwear’; ‘Shoes AND Children’s footwear AND Respectful children’s shoes’
Science Direct	‘Shoes AND Children AND Pediatrics’; ‘Shoes AND Children AND Children’s footwear’; ‘Shoes AND Children’s footwear AND Respectful children’s shoes’
PEDro (Physiotherapy Evidence Database)	‘Shoes AND Children AND Pediatrics’; ‘Shoes AND Children AND Children’s footwear’; ‘Shoes AND Children’s footwear AND Respectful children’s shoes’

## References

[1] Alfageme-García P., Hidalgo-Ruiz S., Rico-Martín S., Calderón-García J.F., Jimenez-Cano V.M., Morán-Cortés J.F., Basilio-Fernández B. (2024). Respectful Children’s Shoes: A Systematic Review. Children.

[2] Gijon-Nogueron G. (2024). Comment on Alfageme-Garcia et al. Respectful Children’s Shoes: A Systematic Review. *Children*
**2024**, *11*, 761. Children.

